# 3D Printing a Clinically Available Solution From Plaster‐Based Positives in Superficial Radiation Therapy

**DOI:** 10.1002/jmrs.70098

**Published:** 2026-07-13

**Authors:** Rory Hartley, Linda Bell, Toby Lowe, Jeremy Booth, Sarah Bergamin, Dasantha Jayamanne, Ryan Brown

**Affiliations:** ^1^ Northern Sydney Cancer Centre, Radiation Oncology Department Royal North Shore Hospital St Leonards New South Wales Australia; ^2^ Institute of Medical Physics, School of Physics University of Sydney Camperdown New South Wales Australia; ^3^ Sydney Medical School University of Sydney Sydney New South Wales Australia

## Abstract

**Introduction:**

This study compares two techniques for creating lead shielding masks to replace the current process for superficial/orthovoltage (SXT) facial radiotherapy: Plaster Cast Positive Mould (PCPM), Computed Tomography (CT) Scanned 3D Print (CT3DP), and Optical Scan 3D Print (OS3DP). The aim is to determine the most accurate and practical method, particularly where PCPM facilities are unavailable.

**Methods:**

From July 2022 to January 2023, twelve patients underwent facial SXT treatment using all three techniques. PCPM used alginate impressions reinforced with plaster. OS3DP employed the Skanect Structure Sensor for optical scanning and PLA 3D printing. CT3DP used CT imaging processed via Varian Eclipse and Adaptiiv 3D Bolus software. Accuracy was retrospectively assessed through surface mesh comparisons, manual point measurements, and blind preference testing.

**Results:**

CT3DP showed the highest accuracy, with a mean maximum deviation of 14.8 mm and average surface difference of 2.6 mm, compared to 17.0 mm and 3.4 mm for OS3DP. Manual measurements highlighted better alignment at the eyebrows with CT3DP. In blind testing, 81.4% of participants preferred CT3DP over OS3DP. PCPM was the most time‐intensive, taking 16 h to complete, vs. six hours for the 3D printing methods.

**Conclusion:**

CT3DP offers greater accuracy, efficiency, and clinical preference compared to OS3DP and PCPM. Despite requiring a CT scan and associated imaging dose, its precision and reproducibility make it a strong candidate to replace PCPM in routine clinical practice.

## Graphical abstract

This study compares two techniques for creating lead shielding masks to replace the current process for superficial/orthovoltage facial radiotherapy: Plaster Cast Positive Mould (PCPM), CT Scanned 3D Print (CT3DP), and Optical Scan 3D Print (OS3DP). CT3DP was found to be more accurate and preferred over OS3DP. CT3DP's precision in contouring and treatment accuracy made it a viable alternative to replace PCPM in clinical practice.

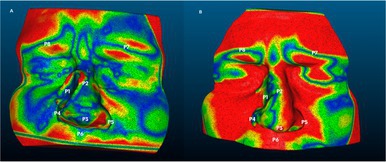

## 1 Introduction

As early as 1990, soon after its introduction into the manufacturing industry, stereolithographic 3‐Dimensional (3D) printing was used to create polymer models of human skulls and in situ knee joints from computed tomography (CT) images [1]. The 3D printing process has evolved exponentially since its inception, more recently used for “tissue and organ biofabrication; creating prosthetics, anatomical models, and implants; and in pharmaceutical research regarding drug discovery, delivery, and dosing.” [2, 3].

There are three main categories of 3D printing: Stereolithography (SLA), Thermal Inkjet Printing (Bioprinting), and the much more common and inexpensive Fused Deposition Modelling (FDM). An FDM printer utilises a printhead not dissimilar to an inkjet printer, but instead of liquid/gel being applied to a surface membrane, thin thread‐like spools of thermoplastic are extruded through a heated printhead as it moves, building the object in multiple predefined layer thicknesses [4]. FDM printing of 3‐dimensional objects has a variety of useful applications in radiation therapy, as seen in cancer treatment departments across the globe. They range from radiation shielding, patient‐specific boluses, brachytherapy treatment applicators, and immobilisation [5, 6, 7, 8, 9]. To create these customised accessories, accurate patient topography must be obtained, typically collected through the use of a CT scanner and subsequent image thresholding [7, 10]. This process generates an external contour which substitutes as a delineation of the patient's skin, and can be used in a treatment planning system (TPS) to create a highly conformal external bolus. Exporting this structure into commercially available 3D modelling software enables FDM 3D printing of the custom bolus and later clinical use.

Optical scanning offers a different method to obtain a 3D topographical surface render but can vary in cost, complexity, and precision, ranging from consumer‐grade 3D camera systems [9, 11, 14] and inexpensive smartphone technologies, [12, 13] to costly and professional metrology‐grade scanners [15]. The accuracy and precision of these systems are directly correlated to the cost and quality of these methods.

In addition to the clinical use of 3D printed bolus in megavoltage radiotherapy, these technologies can also be used for the formation of lead shielding in superficial/orthovoltage (SXT) radiation treatment [8, 16] to treat non‐melanoma skin cancers and keloids [17]. For SXT treatment, the normal clinical procedure is to create a high‐quality negative impression mould of the patient's face with dental alginate material, reinforced with Plaster of Paris, with the clinician's delineation of the treatment area directly transferred across in this process. Once rigid, the mould is filled with fluid dental stone and allowed to set, creating a positive model of the patient's face. Depending on the energy of photons prescribed, a sheet of lead of predefined thickness is manually shaped over the model, and the area of treatment is carved from this lead sheet. This offers collimation of the SXRT beam to the target area and shielding of nearby OARs. Alternatively, an external skin surrogate can be exported from a CT scan, which is refined using 3D modelling software, allowing for a high‐resolution positive model of the patient's face to be FDM printed. Consumer‐grade optical scanning has also been shown to quickly acquire an image of a patient's face, be processed through 3D modelling software, and have an adequate positive model FDM printed [8].

Due to plaster cast positive mould (PCPM) facilities being removed from clinical practice in the local department, the need to find a replacement technique was born of necessity due to a general trend of fewer resources and a lack of manual skill needed for the classic plaster technique. Initial results from the optical scanned 3D print (OS3DP) were promising but lacked crucial detail around complex structures such as the ala nasi and orbits, so an alternative method was required. This paper will characterise and compare the advantages and disadvantages of the PCPM, CT‐scanned 3D print (CT3DP), and the OS3DP techniques and technologies to create accurate lead shielding for use in SXT radiotherapy.

## 2 Methods and Materials

### 2.1 Mask Manufacturing Methods and Patient Cohort

**FIGURE 1:**
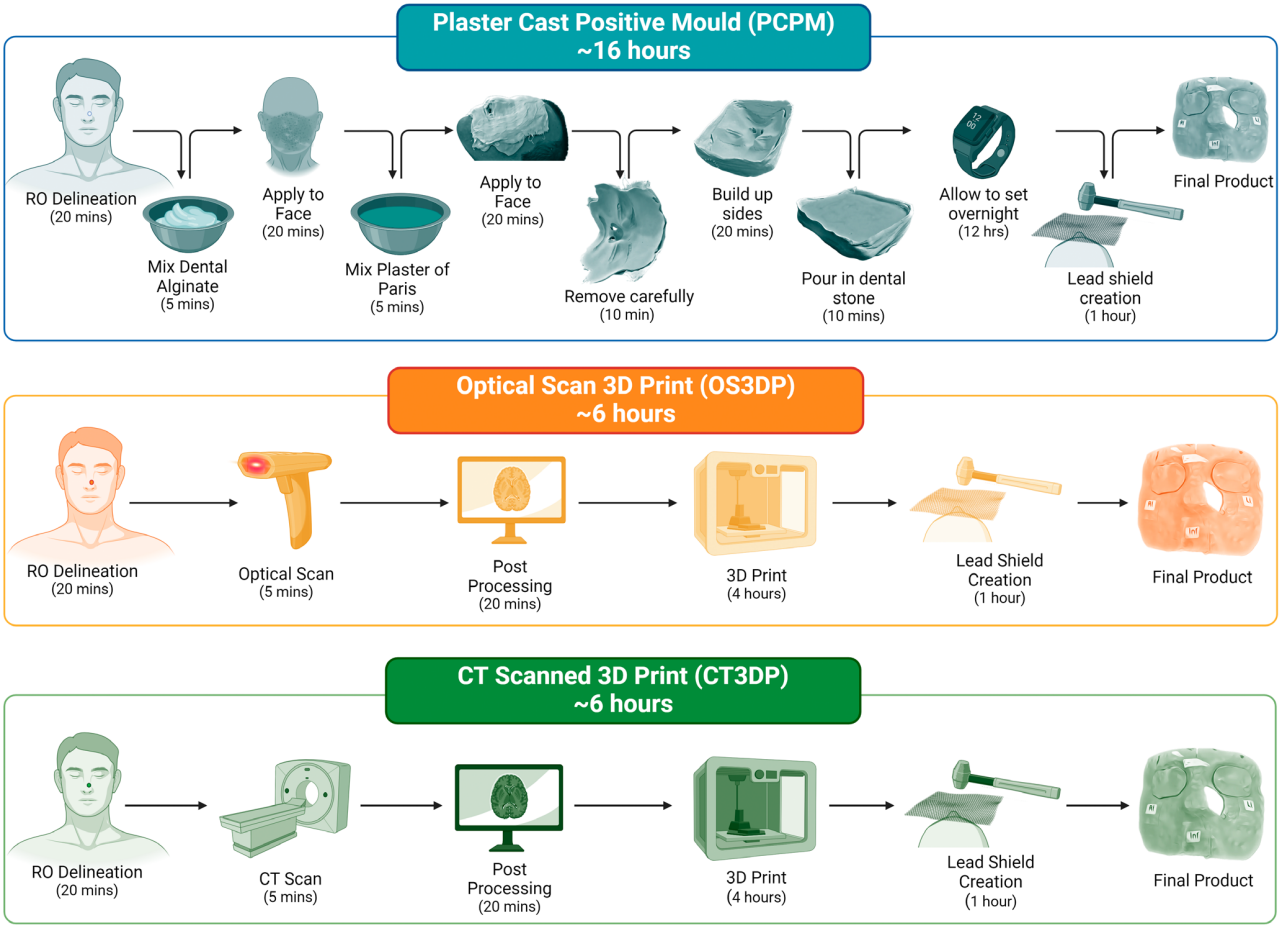
Process and time taken to complete each step of the mask‐making techniques. The Blue represents the Plaster Cast Positive Mould (PCPM), the Orange the Optical Scan 3D Print (OS3DP), and the Green the CT Scanned 3D Print (CT3DP).

Between July 2022 and January 2023, 12 patients aged between 60 to 99 years old receiving facial 100 kV SXT treatment had a positive model generated using each of the PCPM, CT3DP, and OS3DP techniques. Figure 1 summarises the workflow for each approach. 3D print settings are available in the Supporting Information.

### 2.2 Plaster Cast Positive Mould (PCPM)

Following the PTV delineation in thick felt tip pen and oil‐based pencil by a Radiation Oncologist (RO), the patient was positioned head‐first supine (HFS) and asked to close their eyes and breathe through their nose. An initial negative impression was taken using Jeltrate Alginate Impression Material (Dentsply Sirona, North Carolina, USA). Due to its fragile state after setting, the alginate impression was reinforced by strips of Gypsona Plaster of Paris (BSN Medical BmbH, Hamburg, Germany). Post drying, the negative impression was carefully removed from the patient's face, care taken not to damage the fragile alginate. After drying and further reinforcement, the cavity was filled with Yellowstone Dental Stone (Ainsworth Dental Co, Sydney, Australia) and left to solidify for 12 h. After the removal of the Plaster of Paris and Alginate, the positive retained the Planning Target Volume (PTV) that was transferred across and was ready for the shaping of the lead shielding.

### 2.3 Optical Scan 3D Print (OS3DP)

In each case, the patient was positioned in HFS and asked to close their eyes, and the PTV was marked on by the RO using a dark coloured pen. Using the Skanect Structure Sensor and 3D Scanning Software (Occipital, Colorado, USA), a scan was taken of the patient free‐hand with visual feedback on the laptop to assist in maintaining a set distance in two 180‐degree arcs (right to left then left to right); the model reconstructed and then exported to MeshLab (Istituto di Scienza e Tecnologie dell'Informazione, Pisa, Italy) to create the PTV indentation. After this, the file was imported into ideaMaker, a 3D slicing software attached to the Raise3D N2 Plus Printer (Raise3D, California, USA) that automatically generated support structures while providing a set of tools for manual editing.

### 2.4 CT Scanned 3D Print (CT3DP)

**FIGURE 2:**
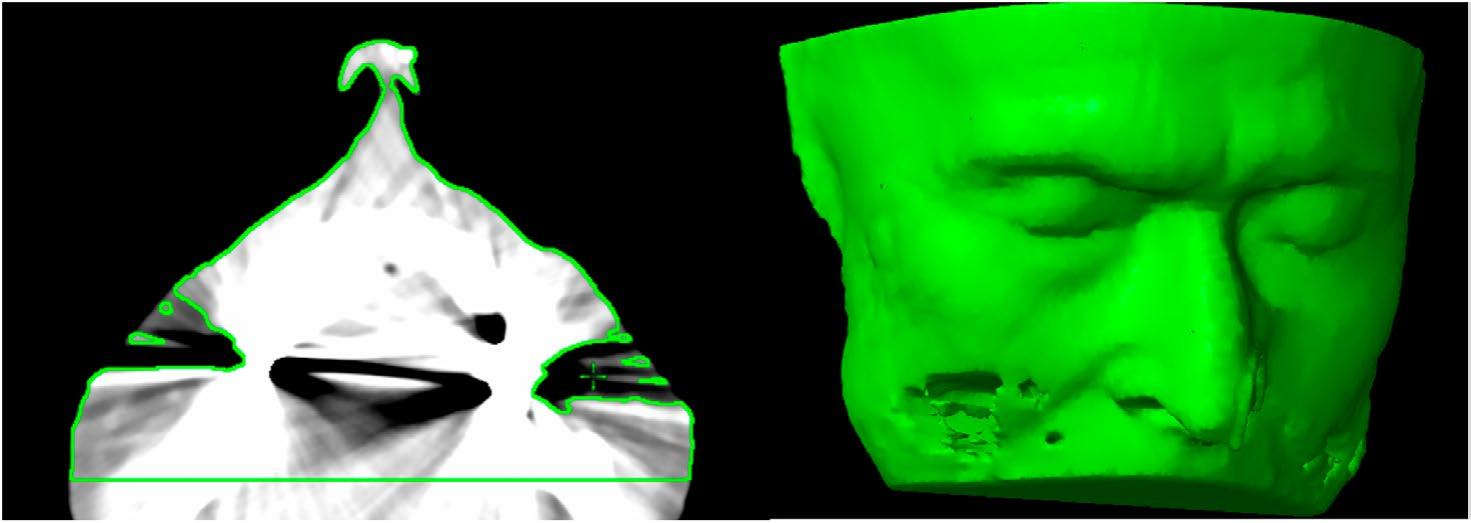
CT scans that have artefact from teeth fillings (left) and the effect on the 3D print (right).

CT images of the patients were acquired using a Philips Brilliance CT BigBore Scanner (Philips Medical Systems, Ohio, USA) with a purpose‐built exam card with 1 mm slices and 1024 × 1024 resolution. In each case, the patient was positioned HFS, taking care to adjust the transverse plane of the teeth by manipulating the patient's head pitch due to the detrimental artefact dental fillings created (Figure 2).

Wire was placed on the RO‐outlined PTV, enabling radiation therapists to transfer this to the exported contour for printing. This data was then imported into the Varian Eclipse Contouring workspace (Version 13.7, Varian Medical Systems, Palo Alto, USA). To create an accurate contour to export and 3D print, a high‐resolution structure was created, and an image threshold of −150 Hounsfield Units (HU) to maximum was applied, post‐processed and enhanced to fill all cavities. The wire was removed from this contour and indented using a 2.0 mm eraser tool to enable an accurate representation of the PTV to be transferred. When exported from Eclipse, each scan must be converted into a single STL file using Adaptiiv 3D Bolus software (Adaptiiv Medical Technologies Inc., Nova Scotia, Canada). This STL file was then imported into the Raise 3D N2 Plus printer. A standardised printing template was created and used 2 shells, a layer height of 0.40 mm and an infill density of 20% to strike a balance between efficiency and strength. The printer used 1.75 mm PLA material with a 0.80 mm diameter nozzle with a target temperature of 220°C on a printer bed held at 70°C.

To ensure compliance with the ALARA (As Low As Reasonably Achievable) principle and to minimise radiation exposure, the mA on the CT Scan Card was adjusted from 225 to 161, which reduced the mGy/cm by 36%. (Table 1). This adjustment was made based on the optimisation of an external contour within the TPS.

**TABLE 1:** Dose reduction achieved with the DoseRight Index reduction.

	Milliampere‐Seconds (mAs)	DoseRight Index	Volume Computed Tomography Dose Index CTDI_vol_ (mGy)	Dose Length Product DLP (mGy/cm)
Standard CT Parameters	225	32	29.1	772.9
New CT Parameters	161	28	18.5	491.2

### 2.5 Time Analysis

A time analysis was conducted to compare the duration of each procedural workflow; all time measurements were recorded using an AnyTime XL‐013 digital stopwatch.

## 3 Analysis

### 3.1 Spatial Accuracy

**FIGURE 3:**
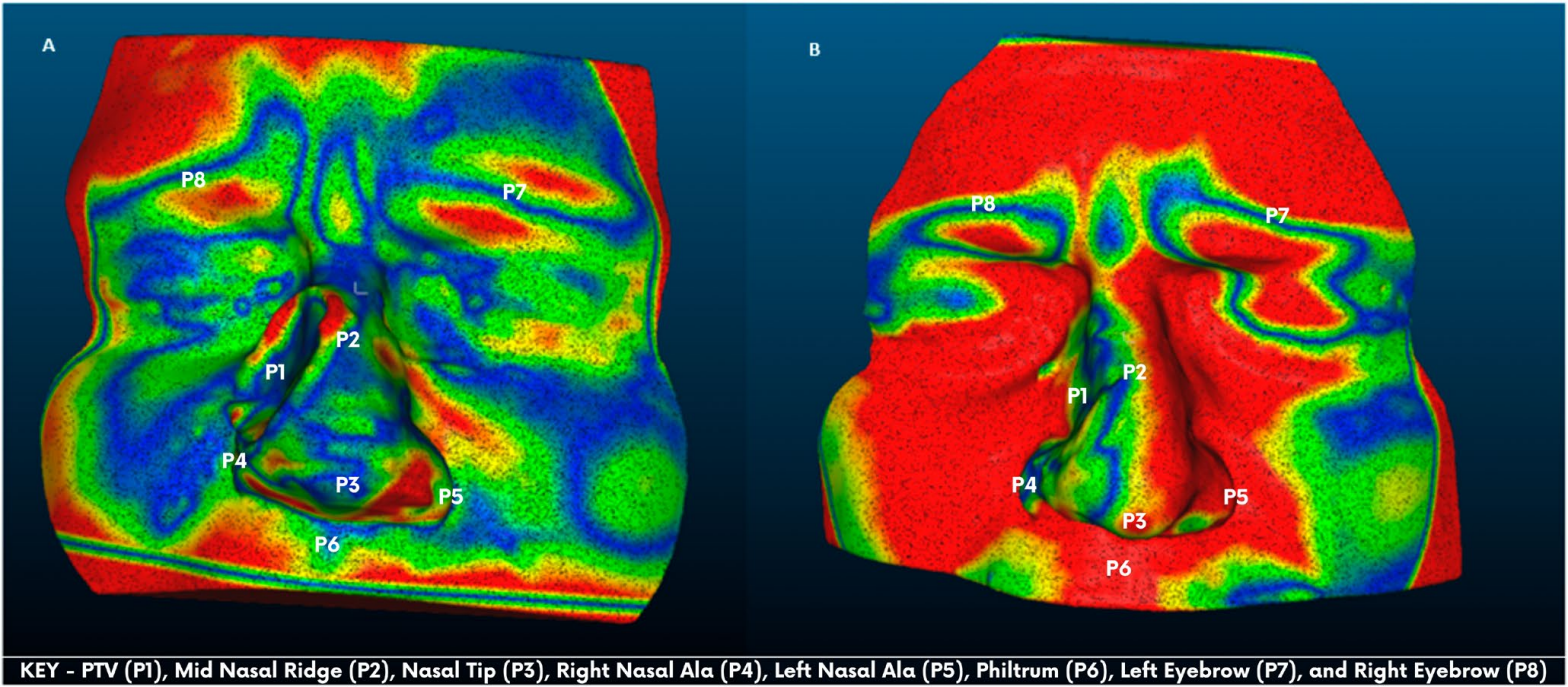
Cloud Compare generated Hausdorff distance colour scales. The Blue represents a distance of < 1 mm, the Green between 1 to 2 mm, and the Red > 2 mm. Exhibit A shows the CT Scanned 3D Print (CT3DP) vs. the Plaster Cast Positive Mould (PCPM), with Exhibit B showing the Optical Scan 3D Print (OS3DP) vs. the PCPM.

A comparison of the surface mesh between the candidate methods and ‘ground truth’ was performed to quantify the accuracy of each approach. To compare the three methods, the plaster positive and the OS3DP and CT3DP positives were CT‐scanned and imported into Eclipse. Bellis et al. [18], found that windowing on CT scans creates a discrepancy of measurements within 3D printing of superficial brachytherapy. To minimise this uncertainty and accurately measure the difference between the plaster positive and 3D prints within the Eclipse contouring workspace, identical image thresholding was used to create consistent delineation of facial contours. To further increase the validity and standardise the software‐based results, the three structures were cropped in Eclipse to 2.0 cm superiorly to the superior orbital margin, 1.0 cm inferiorly to the columella and 3.5 cm posteriorly from the philtrum. These contours were exported into the Adaptiiv 3D Bolus software used in the CT scanning 3D print process to convert the data into a usable STL file. These files were then compared using CloudCompare (Version 2.12.4, https://www.danielgm.net/cc/), freely available 3D point cloud and mesh processing software. The point cloud comparison was registered using eight point‐based alignments. For the comparison, a cloud‐to‐cloud distance computation was performed comparing the CT3DP and OS3DP with reference to the PCPM and standard deviation (σ) calculated. As seen in Figure 3, the resulting scalar fields Hausdorff distance colour scale was generated with a tolerance of 2.0 mm, as well as the maximum distance, average distance, sigma, and maximum error.

### 3.2 Manual Point Measurements

To strengthen reliability, an additional measurement was devised due to observed disparities in lead shielding among the three mask manufacturing methods. All three positives were CT‐scanned and imported into Eclipse. The CT3DP and OS3DP were aligned with the PCPM and Auto Matched by selecting an appropriate Volume of Interest (VOI): 2.0 cm superiorly to the superior orbital margin, 1.0 cm inferiorly to the columella, entire contour laterally, and 3.5 cm posteriorly from the philtrum. Subsequently, these alignments were verified by an impartial third party. Our investigation encompassed eight specific areas of interest, denoted as follows: PTV (P1), Mid Nasal Ridge (P2), Nasal Tip (P3), Right Nasal Ala (P4), Left Nasal Ala (P5), Philtrum (P6), Left Eyebrow (P7), and Right Eyebrow (P8). At each of these designated points, the distance was measured between × and y by a single observer using a virtual ruler and was repeated to understand uncertainty. The same image windowing was used to standardise contour delineation in the planning system (Upper HU: 225, Lower HU: −125).

### 3.3 Blind Comparison Test

In a dedicated room chosen for blind testing, the positives were arranged with the PCPM positioned at the centre and the CT3DP and OS3DP placed on either side in random order. Individually, 27 RTs, 7 Physicists, and 10 Radiation Oncology Registrars and Staff Specialists were then invited into the room with the task of assessing which 3D print they judged most closely resembled the gold standard PCPM based on look and feel.

## 4 Results

### 4.1 Time Analysis

Assuming no delays throughout the procedure, the overall duration for completing the OS3DP and CT3DP processes, including image acquisition, post‐processing, and FDM printing, was approximately six hours. In contrast, the total time needed for the PCPM method was notably longer, totalling 16 h, which encompassed the stages of negative impression, plaster curing, and positive setting. Regarding the patient's time commitment, the OS3DP and CT3DP methods required approximately 30 min in the department, while the PCPM method required around 90 min (see Figure 1), including time to clean up residue left after the PCPM technique.

### 4.2 Surface Mesh Comparison

The cloud‐to‐cloud comparison computed a mean max difference of the 12 patients as 14.8 mm (σ 3.7) for the CT3DP and 17 mm (σ 8.4) for the OS3DP. The average distance between the two mesh clouds was 2.6 mm (σ 1.0) for the CT3DP and 3.4 mm (σ 2.1) for the OS3DP. Qualitative differences were observed between the cloud comparisons using the associated colour scales representative of the differential Hausdorff distances for each match. In this case, the CT3DP was more closely aligned with the PCPM than with the OS3DP.

### 4.3 Manual Measurements

**FIGURE 4:**
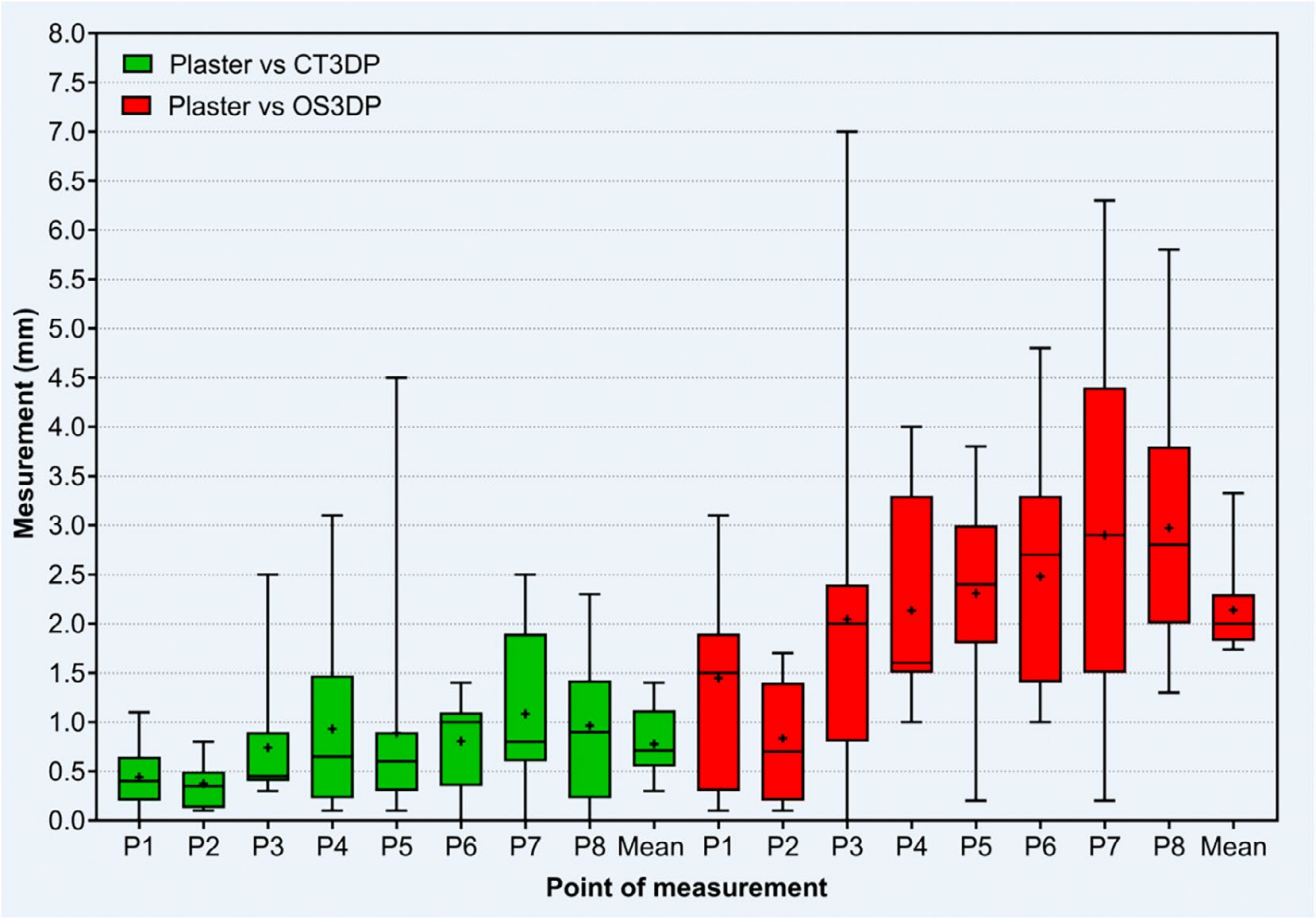
Point measurement mean, and range variation between CT Scanned 3D Print (CT3DP) and Plaster Cast Positive Mould (PCPM) (Green) and Optical Scan 3D Print (OS3DP) and PCPM (Red).

Out of the eight points that were measured, the most significant disparity in means was observed for the Left Eyebrow (P7) for the CT3DP, measuring at 1.1 mm (σ 0.8), and Right Eyebrow (P8) for the OS3DP, which yielded a measurement of 3.0 mm (σ 1.3). The smallest difference was identified at Mid Nasal Ridge (P2) for both methods, where the CT3DP exhibited a difference of 0.4 mm (σ 0.2), and the OS3DP showed a difference of 0.8 mm (σ 0.6) (Figure 4).

### 4.4 Blind Comparison Test

**FIGURE 5:**
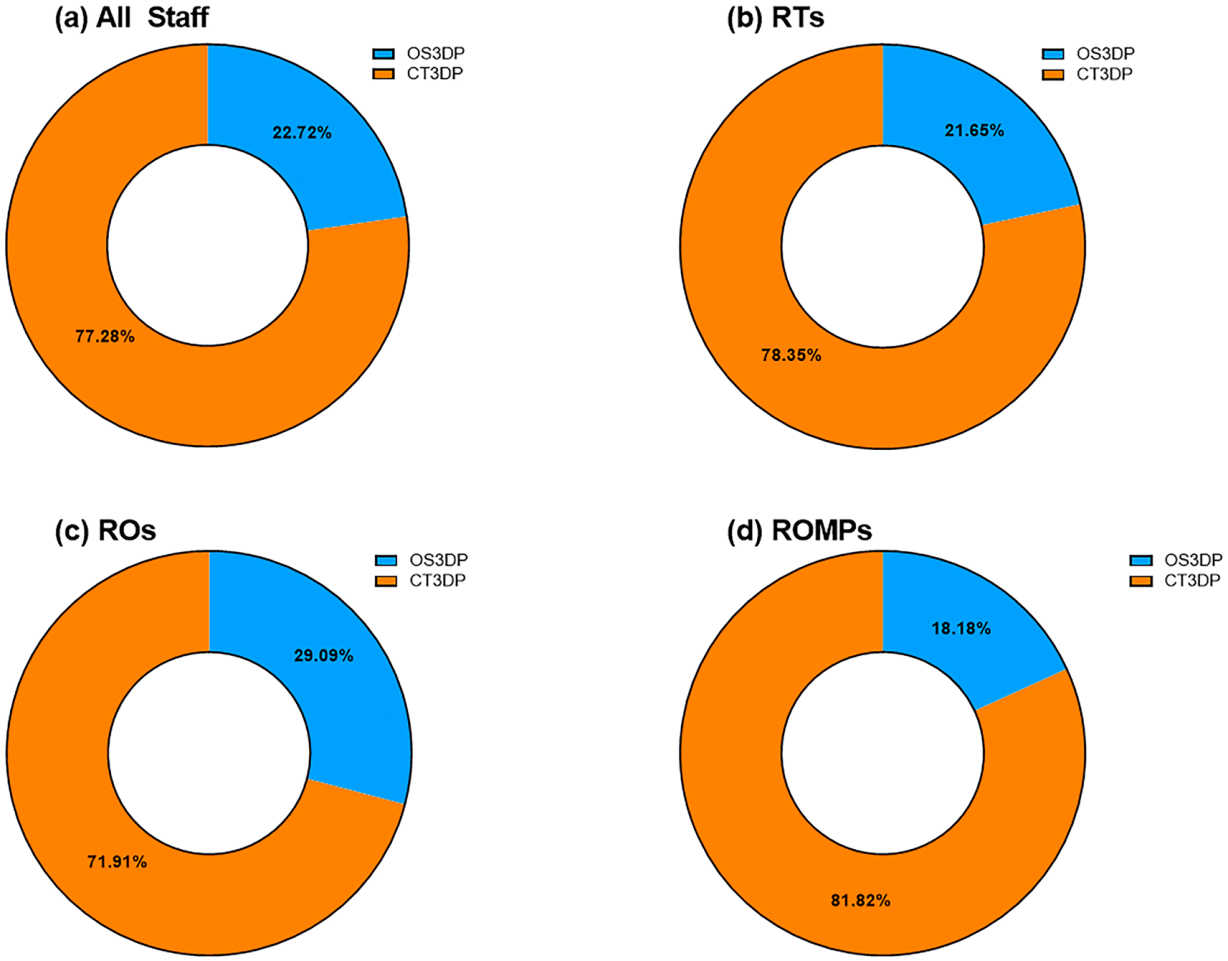
Blind Comparison Test frequency distribution of the CT Scanned 3D Print (CT3DP) and Optical Scan 3D Print (OS3DP) to the Plaster Cast Positive Mould (PCPM).

Of the 11 masks compared, the majority agreed that the CT3DP more closely resembled the gold standard, with 81.4% choosing the CT3DP over the OS3DP, as shown in Figure 5.

## 5 Discussion

Historically, CT would not be used for superficial radiotherapy. However, due to the success of the technique in other tumour sites, the SXT multi‐disciplinary team (MDT) made the decision to extend low‐dose CT scanning to patients requiring intricate lead face shielding. The utilisation of CT‐based 3D printing had already proven effective in creating 3D printed boluses [15] for patients with breast, chest wall, sarcoma, and head and neck cancer. While it is important to note that this new approach results in additional radiation dose compared to the standard method, it was justified based on mask production accuracy, improved workflow, patient comfort and the lower stochastic risk of lifetime cancer for elderly patients, as outlined in the age category risk limits discussed by the Radiation Protection of Patients Unit of the International Atomic Energy Agency [19].

In this study, we modified a standard Brain CT scan template for 3D printing. This customisation optimised the CT template for our 3D printing needs, including a 1 mm slice thickness and an increased resolution for the reconstruction grid (1024 × 1024 pixels). Our approach involved conducting scans from two distinct anatomical landmarks, extending from the eyebrows to the lower lip, to ensure that radiation exposure maintained adherence to the ALARA principle.

Increased accuracy of the treatment site was aided by the CT scan, providing ROs with exact tumour depth data, which is a deciding factor in beam energy selection and clearly defines the backscatter factor of underlying tissue density used in the monitor unit calculations for planning. Careful consideration of the transverse plane must also be taken at the time of CT to minimise the impact of the teeth filling artefact, which can negatively affect the accuracy of the treatment site and facial contour delineation in the TPS (Figure 2), thus creating an incorrect 3D print on export.

Another advantage of the CT technique is that the data is saved within the patient's electronic health record, and precise delineation of previous treatments will be available for future use. For patients needing treatment elsewhere on their face, assuming no drastic changes, the same dataset may be used to re‐print a positive or plan megavoltage treatments in the TPS. There is currently no capacity in most modern TPS for superficial radiotherapy to be calculated. Should this become available, a retrospective planning study could be undertaken with the data collected to assess the accuracy of previous manual calculations and to determine if computer‐planned treatments provide similar outcomes and toxicity for patients.

At the department, over the five‐year period from June 2018 to June 2023, a total of 239 patients with an average age of 74.7 years underwent SXT radiotherapy. It is well documented that as elastin biosynthesis begins to decline through ageing, the facial skin loses elasticity as the elastic fibre network disintegrates [20]. The alginate material exerts pressure on the skin in regions such as the eyebrows, cheeks, and nasolabial folds, leading to the formation of an imprecise PCPM, although this may mimic the weight of the lead shielding. The accurate positioning of the patient is a critical factor in SXT mark‐ups, and in the case of the PCPM and CT3DP techniques, the patient must lie supine/inclined, which can be challenging for older patients. Conversely, the OS3DP permits capturing images from various angles, affording enhanced flexibility in patient positioning, although changing the effect of gravity on facial skin.

Obtaining images more swiftly through the OS3DP^8^ and CT3DP methods not only eases the strain on resources but also contributes to a more comfortable experience for patients. This approach reduces their physical time in the department from 90 min to 30 min, and it minimises the sense of claustrophobia that some patients may experience, [21] which is more pronounced with the PCPM technique. This reduced time may also allow patients to start treatment sooner, which is important for palliation and local response/control.

PCPM application is limited by the need for staff training, a constant supply of specialised and relatively expensive materials, and a dedicated plaster waste facility within the radiation therapy department. Higher‐cost, metrology‐grade scanners, such as the Artec Leo used by Crowe et al., offer superior accuracy and resolution compared to the optical scanner used in this study and would be the preferred option [6]. However, many departments may be unable to accommodate the upfront expense of the required optical scanning hardware and software for OS3DP, which may restrict its implementation, and would rather opt for a lower cost scanner as demonstrated by Shin et al. [11]. Additionally, after acquiring the scan, complex processing is completed in specially designed software programs (Skanect, Meshmixer, Ideamaker) which all require separate subscriptions and a high‐performance computer which may potentially limit their use. Contrary to this, every radiotherapy department has access to a CT scanner and TPS. Even though this study used 3D Bolus software to convert CT data, open‐source software can also create the necessary STL files for export and print [8].

Due to the small amount of clinical training needed to operate the scanner and the fact that it is not required to be in a specialised facility like the PCPM and CT3DP, OS3DP would free up both CT staff and resources. However, the OS3DP and CT3DP are limited by the requirement to have access to a 3D Printer or the ability/resources to outsource to a 3D printing company. During the process, the RTs had some issues connecting the scanner to the iPad, computer, and the internet in some instances and were therefore unable to take one set of images for a patient in this study (Patient 3).

The PCPM method may not be suitable for certain SXT patients due to the requirement of applying non‐sterile materials to an open wound. For instance, one patient with an open cavity and facial tenderness was unable to undergo the PCPM method. However, this patient successfully received a mask created using both the CT3DP and OS3DP techniques, which involve minimal physical contact. Additionally, some optical scanning systems necessitate the use of high‐intensity flashing lights to capture contour information. This approach is both uncomfortable for patients and unsuitable for individuals with medical conditions such as epilepsy.

From a qualitative perspective, CloudCompare proved to be quite valuable in the context of method comparison. Quantitatively, however, the reliability and consistency of the point cloud comparisons were unreliable. After registering the scans using the 9‐point‐based alignments, an attempt was made to fine‐tune the registration using the iterative closest point algorithm. This approach was chosen due to its capability to utilise six degrees of freedom and its accuracy independent of shape representation, as described by Besl and McKay [22]. However, the rigid bounding box and its inability to define a specific volume of interest caused issues. Differences in size, shape, and angle among the scans made the automatic registration tool less accurate, reflected in the higher standard deviation for these results compared to the manual point‐based method. Consequently, the point‐based method of registration was preferred. It's worth noting that variations in image registration methods and software analysis introduce inherent uncertainties that cannot be fully addressed in this study, representing a limitation in our analysis within CloudCompare.

There were notable variations in the shape of the lead shielding among the three methods. Unfortunately, this study was unable to directly compare the lead shielding across the PCPM, OS3DP, and CT3DP due to discrepancies in the rigid positive that rendered the masks incompatible across different forms, and due to the retrospective nature of this study, the lead shielding could not be tested on the patients themselves. The CloudCompare matches demonstrated that, given the intricate nature of facial structures, unless the contours were perfectly matched, the lead shield would inevitably not fit. This was physically confirmed by the staff and supported by the results of the blind comparison test (Figure 5).

## 6 Conclusion

In conclusion, CT3DP is the most promising alternative to PCPM for facial lead shielding, providing a balance between accuracy, patient comfort, and workflow efficiency. The OS3DP method shows promise; however, its implementation is constrained by the need for high‐quality and costly hardware, which limits its widespread use. Further research could explore the integration of these techniques into standard clinical practice, particularly for superficial radiotherapy planning in treatment planning systems.

## Ethics Statement

The Northern Sydney Local Health District's Human Research Ethics Committee at the Royal North Shore Hospital authorised this Quality Improvement Project: RNSH QIP 18–2023.

## Conflicts of Interest

The authors declare no conflicts of interest.

## Supporting information


**Data S1:** 3D Printer Settings.

## Data Availability

The data that support the findings of this study are available from the corresponding author upon reasonable request.
