# Supplementary figures and images for: 3D Printing a Clinically Available Solution From Plaster‐Based Positives in Superficial Radiation Therapy
Source: J Med Radiat Sci. 2026 Jul 13:10.1002/jmrs.70098. Online ahead of print. doi: 10.1002/jmrs.70098 (PMC13398848; doi:10.1002/jmrs.70098)

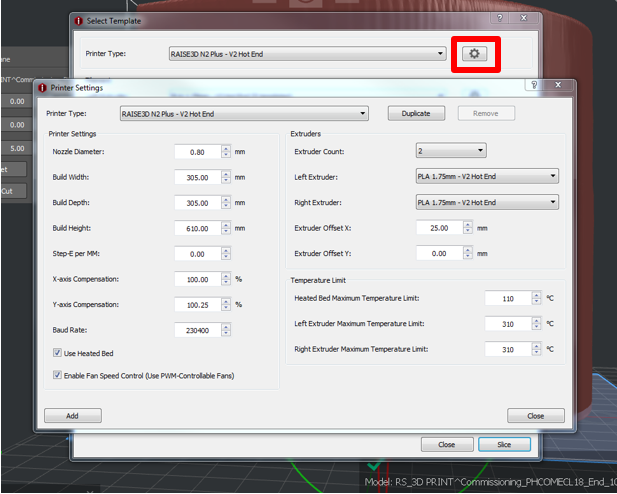

Supplement: Supplementary file 1 — Data S1: 3D Printer Settings. [file JMRS-9999-0-s001.png]
